# Supplementary material for: Diverse microbial communities hosted by the model carnivorous pitcher plant Sarracenia purpurea: analysis of both bacterial and eukaryotic composition across distinct host plant populations
Source: PeerJ. 2019 Feb 18;7:e6392. doi: 10.7717/peerj.6392 (PMC6383556; doi:10.7717/peerj.6392)
Supplement: Figure S1 — Comparison of community composition between the two wetlands populations, Cedarburg and Sapa. Bacterial (top) and Eukaryotic (bottom) families identified within pitcher samples, showing data pooled for the 5 samples from each wetland (shown in Fig. 1) for families representing <0.01% of the total pooled sequences, and sequence numbers are adjusted to the mean of the two populations. [file peerj-07-6392-s001.pdf]

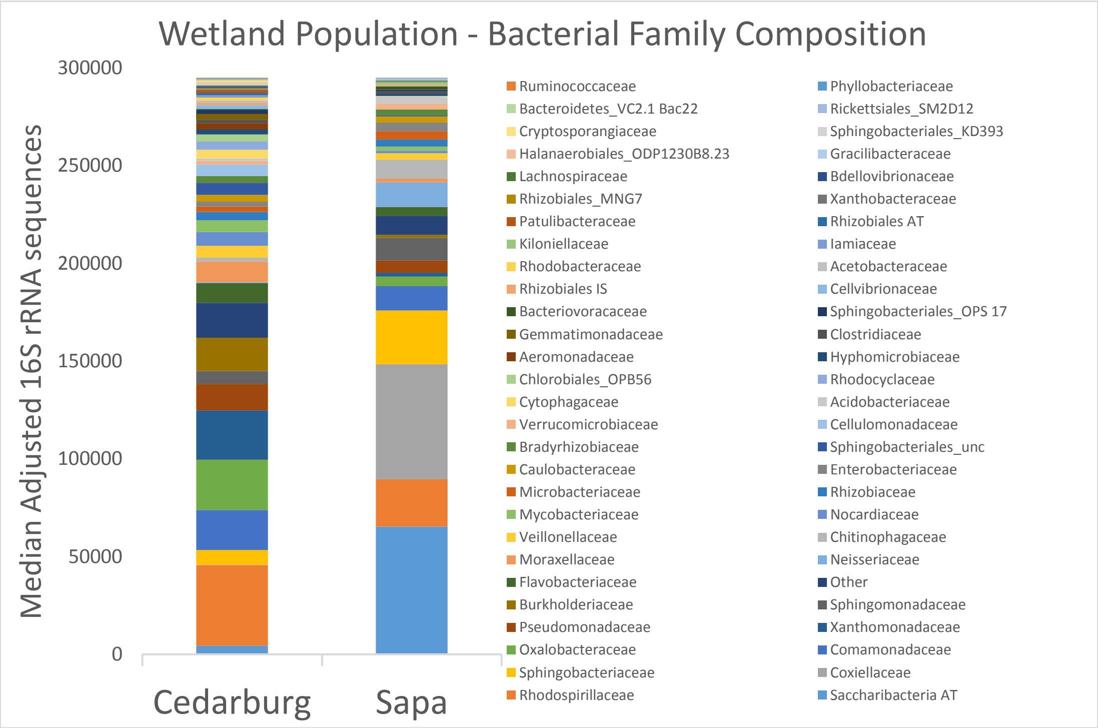

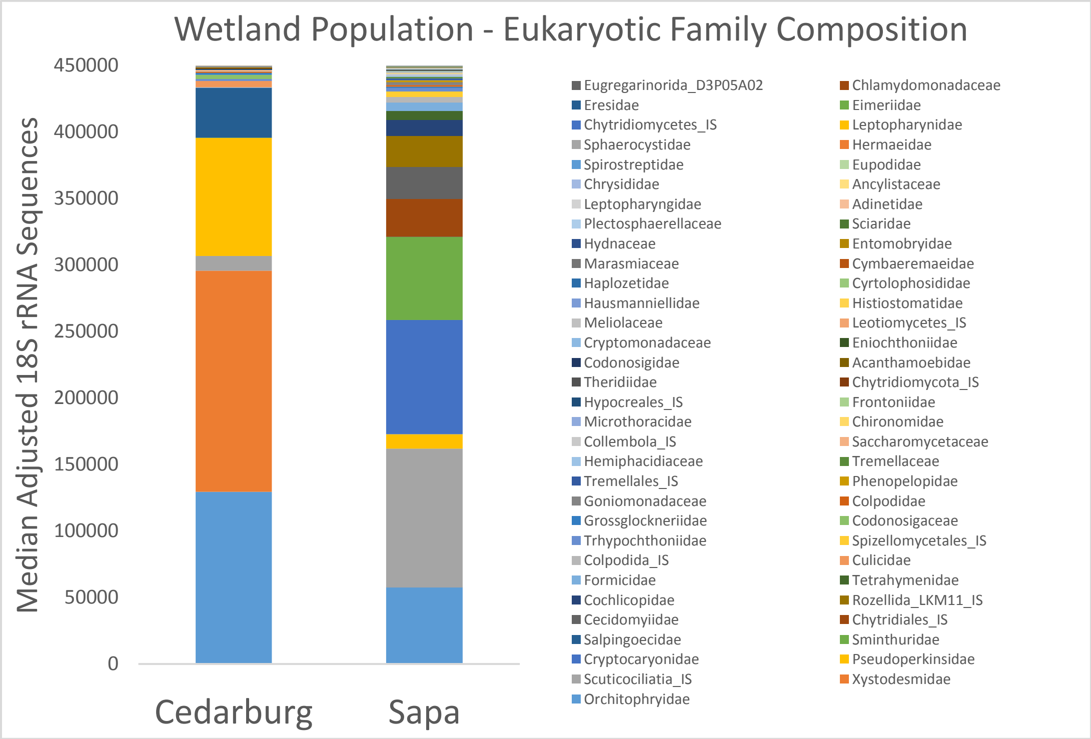

Figure S1. Comparison of community composition between the two wetlands populations, Cedarburg and Sapa. Bacterial (top) and Eukaryotic (bottom) families identified within pitcher samples, showing data pooled for the 5 samples from each wetland (shown in Fig. 1) for families representing > 0.01% of the total pooled sequences, and sequence numbers are adjusted to the mean of the two populations.
