# Supplementary material for: Diverse microbial communities hosted by the model carnivorous pitcher plant Sarracenia purpurea: analysis of both bacterial and eukaryotic composition across distinct host plant populations
Source: PeerJ. 2019 Feb 18;7:e6392. doi: 10.7717/peerj.6392 (PMC6383556; doi:10.7717/peerj.6392)
Supplement: Figure S2 — Heatmap showing bacterial and eukaryotic OTUs common between samples - lower similarity of composition between samples in white and higher similarity in black. Bacterial OTUs are in the upper triangle and eukaryotic OTUs are the lower triangle. The bacterial and eukaryotic datasets use the same color scale but have different minimum and maximum values due to different total OTU abundances between datasets. [file peerj-07-6392-s002.pdf]

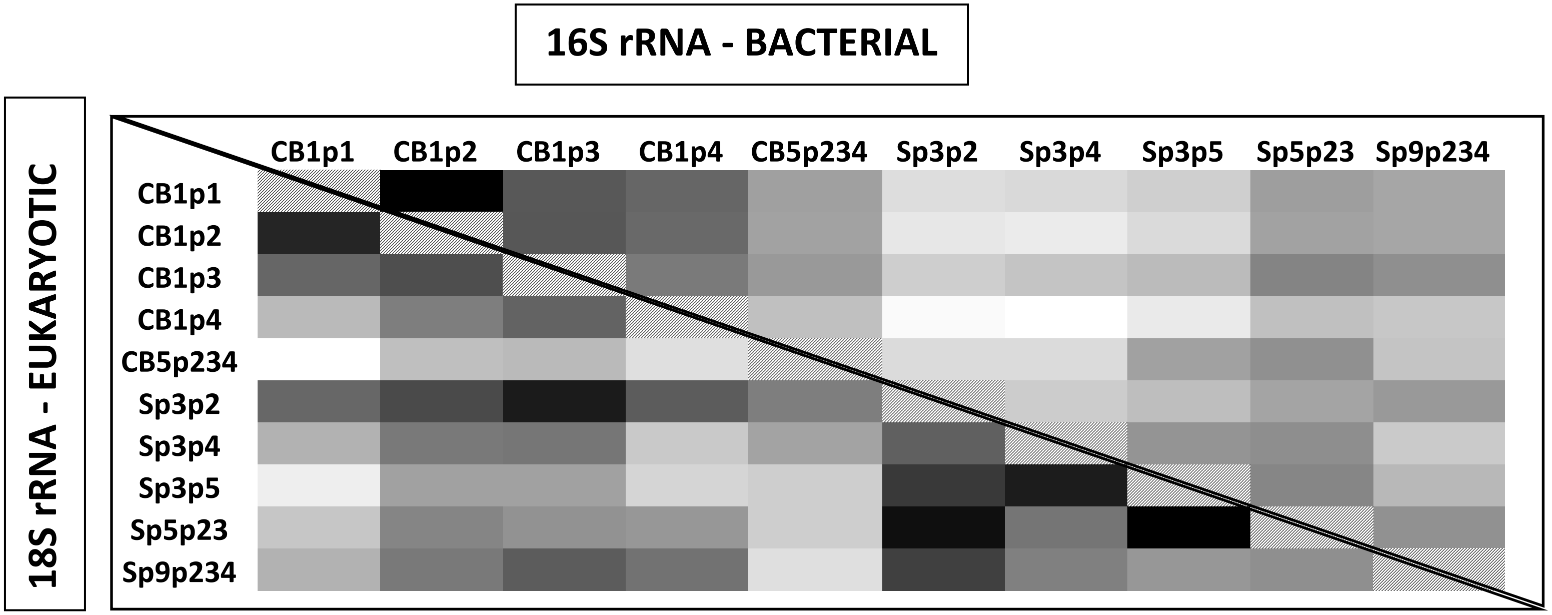

Color Key (Number of Shared OTUs)

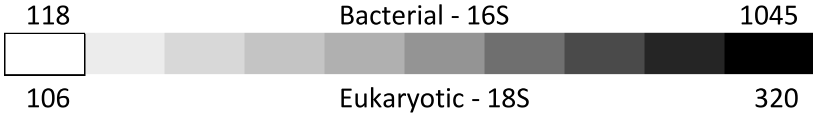

Figure S2: Heatmap showing bacterial and eukaryotic OTUs common between samples - lower similarity of composition between samples in white and higher similarity in black. Bacterial OTUs are in the upper triangle and eukaryotic OTUs are the lower triangle. The bacterial and eukaryotic datasets use the same color scale but have different minimum and maximum values due to different total OTU abundances between datasets.
