# Supplementary material for: Diverse microbial communities hosted by the model carnivorous pitcher plant Sarracenia purpurea: analysis of both bacterial and eukaryotic composition across distinct host plant populations
Source: PeerJ. 2019 Feb 18;7:e6392. doi: 10.7717/peerj.6392 (PMC6383556; doi:10.7717/peerj.6392)
Supplement: Figure S3 — Rarefaction curve of OTUs identified in Cedarburg (CB) and Sapa (Sp) Bog pitcher samples based on sequencing of 16S rRNA (left) and 18S rRNA (right) genes. [file peerj-07-6392-s003.pdf]

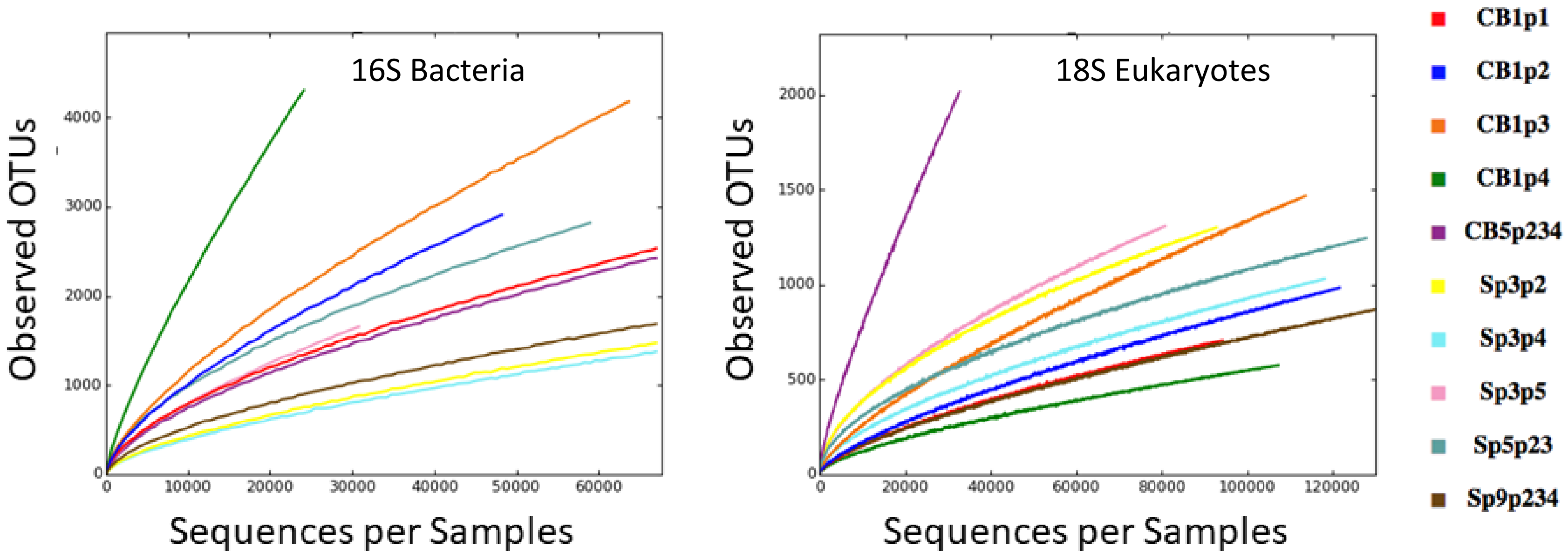

Figure S3. Rarefaction curve of OTUs identified in Cedarburg (CB) and Sapa (Sp) Bog pitcher samples based on sequencing of 16S rRNA (left) and 18S rRNA (right) genes.
