# Supplementary material for: Diverse microbial communities hosted by the model carnivorous pitcher plant Sarracenia purpurea: analysis of both bacterial and eukaryotic composition across distinct host plant populations
Source: PeerJ. 2019 Feb 18;7:e6392. doi: 10.7717/peerj.6392 (PMC6383556; doi:10.7717/peerj.6392)
Supplement: Table S1 — Bacterial OTUs from 16S rRNA sequencing analysis including only taxa which were identified to genus level (those OTUs identified as Ambiguous taxa, uncultured bacteria or without genus-level identifications were omitted). OTUs with only a single representative across all samples were also omitted. Taxa are listed from most to least abundant. [file peerj-07-6392-s004.pdf]

Table S1. Bacterial OTUs from 16S rRNA sequencing analysis including only taxa which were identified to genus level (those OTUs identified as Ambiguous taxa, uncultured bacteria or without genus-level identifications were omitted). OTUs with only a single representative across all samples were also omitted. Taxa are listed from most to least abundant.

				Pitcher Plant Sample										
Class	Order	Family	Genus	CB1p1	CB1p2	CB1p3	CB1p4	CB5p234	Sp3p2	Sp3p4	Sp3p5	Sp5p23	Sp9p234	sum
Gammaproteobacteria	Legionellales	Coxiellaceae	*Rickettsiella*	1	2	0	1	2	3	60194	497	1038	2	61740
Alphaproteobacteria	Rhodospirillales	Rhodospirillaceae	*Azospirillum*	686	527	10513	485	11	3	2	7	16494	8201	36929
Sphingobacteriia	Sphingobacteriales	Sphingobacteriaceae	*Pedobacter*	455	302	873	103	16	19242	279	55	760	1077	23162
Betaproteobacteria	Burkholderiales	Oxalobacteraceae	*Duganella*	9060	5734	2660	40	1357	280	117	29	129	35	19441
Gammaproteobacteria	Pseudomonadales	Pseudomonadaceae	*Pseudomonas*	3336	1991	3475	1309	2819	233	1335	1666	3046	218	19428
Betaproteobacteria	Burkholderiales	Burkholderiaceae	*Paraburkholderia*	0	1	0	1	16051	98	41	140	23	17	16372
Sphingobacteriia	Sphingobacteriales	Sphingobacteriaceae	*Mucilaginibacter*	77	39	3123	20	2006	324	982	5764	408	21	12764
Gammaproteobacteria	Pseudomonadales	Moraxellaceae	*Alkanindiges*	9	10	14	7	9632	6	79	518	1183	65	11523
Betaproteobacteria	Neisseriales	Neisseriaceae	*Aquitalea*	0	0	0	0	1	1577	5715	1471	2141	177	11082
Flavobacteriia	Flavobacteriales	Flavobacteriaceae	*Flavobacterium*	324	219	8432	533	24	123	7	15	111	324	10112
Alphaproteobacteria	Sphingomonadales	Sphingomonadaceae	*Sphingomonas*	633	514	717	498	337	1563	1451	1269	921	864	8767
Gammaproteobacteria	Xanthomonadales	Xanthomonadaceae	*Rhodanobacter*	6	8	2	23	6284	1	1198	781	77	26	8406
Actinobacteria	Corynebacteriales	Mycobacteriaceae	*Mycobacterium*	1321	943	2534	137	865	8	95	506	1294	297	8000
Negativicutes	Selenomonadales	Veillonellaceae	*Pelosinus*	18	11	125	1	4958	127	936	2	786	144	7108
Alphaproteobacteria	Rhizobiales	Rhizobiaceae	*Kaistia*	218	214	2417	374	464	44	97	249	1629	563	6269
Gammaproteobacteria	Xanthomonadales	Xanthomonadaceae	*Tahibacter*	3642	2319	25	2	0	3	1	0	0	1	5993
Actinobacteria	Corynebacteriales	Nocardiaceae	*Rhodococcus*	1073	885	735	2821	32	2	1	0	10	0	5559
Alphaproteobacteria	Sphingomonadales	Sphingomonadaceae	*Novosphingobium*	27	32	10	4	58	8	215	4203	522	10	5089
Actinobacteria	Micrococcales	Microbacteriaceae	*Leifsonia*	47	33	31	52	956	0	807	2294	532	157	4909
Betaproteobacteria	Rhodocyclales	Rhodocyclaceae	*Denitratisoma*	48	33	3746	0	94	35	1	8	42	0	4007
Flavobacteriia	Flavobacteriales	Flavobacteriaceae	*Chryseobacterium*	14	1	47	2	21	3685	28	27	7	3	3835
Actinobacteria	Micrococcales	Cellulomonadaceae	*Oerskovia*	1143	636	30	1948	6	0	0	2	9	0	3774
Alphaproteobacteria	Rhizobiales	Bradyrhizobiaceae	*Rhodopseudomonas*	1082	896	174	273	8	2	0	0	0	1230	3665
Betaproteobacteria	Burkholderiales	Comamonadaceae	*Delftia*	23	25	220	1	149	878	8	10	346	1677	3337
Verrucomicrobiae	Verrucomicrobiales	Verrucomicrobiaceae	*Prosthecobacter*	45	46	41	31	6	0	0	0	90	2964	3223
Gammaproteobacteria	Aeromonadales	Aeromonadaceae	*Aeromonas*	49	24	3049	6	16	0	0	0	1	3	3148
Betaproteobacteria	Burkholderiales	Comamonadaceae	*Variovorax*	776	506	61	167	284	507	152	257	85	68	2863
Gemmatimonadetes	Gemmatimonadales	Gemmatimonadaceae	*Gemmatimonas*	1289	1148	19	234	0	0	1	0	0	0	2691
Acidobacteria	Acidobacteriales	Acidobacteriaceae	*Terriglobus*	1	0	0	0	823	3	704	984	54	73	2642
Gammaproteobacteria	Enterobacteriales	Enterobacteriaceae	*Serratia*	2	0	7	1	2100	126	87	159	109	38	2629
Betaproteobacteria	Burkholderiales	Oxalobacteraceae	*Janthinobacterium*	1073	925	220	8	187	13	48	21	18	0	2513

Actinobacteria	Corynebacteriales	Nocardiaceae	*Gordonia*	30	21	320	688	33	30	260	109	516	481	2488
Deltaproteobacteria	Bdellovibrionales	Bacteriovoracaceae	*Bacteriovorax*	73	50	197	26	0	331	4	1	1442	36	2160
Acidobacteria	Acidobacteriales	Acidobacteriaceae	*Granulicella*	0	0	0	0	350	0	503	895	194	0	1942
Gammaproteobacteria	Cellvibrionales	Cellvibrionaceae	*Cellvibrio*	998	660	21	215	2	0	0	0	26	0	1922
Betaproteobacteria	Burkholderiales	Oxalobacteraceae	*Massilia*	157	174	676	2	126	211	9	13	425	13	1806
Actinobacteria	Micrococcales	Cellulomonadaceae	*Cellulomonas*	262	228	177	1023	0	0	0	0	0	0	1690
Betaproteobacteria	Burkholderiales	Oxalobacteraceae	*Undibacterium*	42	19	773	0	58	48	10	329	295	30	1604
Alphaproteobacteria	Rhizobiales	Rhizobiaceae	*Rhizobium*	43	26	92	39	96	674	117	57	255	204	1603
Sphingobacteriia	Sphingobacteriales	Chitinophagaceae	*Ferruginibacter*	0	1	16	18	134	0	861	24	338	171	1563
Betaproteobacteria	Burkholderiales	Comamonadaceae	*Acidovorax*	529	265	369	23	107	70	37	16	50	42	1508
Betaproteobacteria	Burkholderiales	Comamonadaceae	*Rhizobacter*	28	25	13	0	67	26	13	126	245	955	1498
Alphaproteobacteria	Sphingomonadales	Sphingomonadaceae	*Sphingopyxis*	630	440	57	89	15	2	5	125	98	34	1495
Clostridia	Clostridiales	Clostridiaceae	*Clostridium*	14	256	12	1105	0	0	30	2	3	49	1471
Actinobacteria	Micrococcales	Microbacteriaceae	*Salinibacterium*	441	282	53	198	14	134	29	111	89	89	1440
Alphaproteobacteria	Rhodospirillales	Rhodospirillaceae	*Ferrovibrio*	136	127	1150	5	0	0	0	0	0	0	1418
Betaproteobacteria	Burkholderiales	Comamonadaceae	*Hydrogenophaga*	751	460	95	102	0	0	1	0	0	5	1414
Alphaproteobacteria	Caulobacterales	Caulobacteraceae	*Phenylobacterium*	437	352	21	85	176	0	2	21	246	41	1381
Negativicutes	Selenomonadales	Veillonellaceae	*Anaerospora*	18	10	463	1	4	1	5	0	112	746	1360
Alphaproteobacteria	Rhodobacterales	Rhodobacteraceae	*Rhodovulum*	671	461	43	121	5	0	1	1	13	11	1327
Acidimicrobiia	Acidimicrobiales	Iamiaceae	*Iamia*	563	454	72	106	8	0	0	1	116	0	1320
Alphaproteobacteria	Sphingomonadales	Kiloniellaceae	*Kiloniella*	0	0	0	0	0	173	506	500	103	15	1297
Gammaproteobacteria	Enterobacteriales	Enterobacteriaceae	*Morganella*	0	0	0	0	0	1072	0	6	187	27	1292
Alphaproteobacteria	Caulobacterales	Caulobacteraceae	*Caulobacter*	19	16	5	367	17	15	15	16	715	21	1206
Alphaproteobacteria	Rhizobiales	Rhizobiales IS	*Rhizomicrobium*	58	37	157	19	400	0	3	79	450	2	1205
Alphaproteobacteria	Rhizobiales	Hyphomicrobiaceae	*Devosia*	415	310	174	247	13	1	0	0	0	14	1174
Alphaproteobacteria	Sphingomonadales	Sphingomonadaceae	*Polymorphobacter*	549	395	140	0	3	0	0	0	0	14	1101
Betaproteobacteria	Burkholderiales	Burkholderiaceae	*Chitinimonas*	0	1	0	0	0	0	0	0	1065	0	1066
Alphaproteobacteria	Rhizobiales	Bradyrhizobiaceae	*Bosea*	75	60	150	48	0	217	42	7	40	370	1009
Alphaproteobacteria	Caulobacterales	Caulobacteraceae	*Asticcacaulis*	18	4	62	0	63	3	108	415	225	71	969
Thermoleophilia	Solirubrobacterales	Patulibacteraceae	*Patulibacter*	421	186	335	10	0	1	0	0	0	0	953
Verrucomicrobiae	Verrucomicrobiales	Verrucomicrobiaceae	*Verrucomicrobium*	450	410	36	43	0	0	0	0	0	0	939
Alphaproteobacteria	Caulobacterales	Caulobacteraceae	*Brevundimonas*	218	201	237	196	10	61	0	3	0	12	938
Betaproteobacteria	Burkholderiales	Comamonadaceae	*Aquabacterium*	151	84	85	13	12	1	0	15	429	0	790
Deltaproteobacteria	Bdellovibrionales	Bdellovibrionaceae	*Bdellovibrio*	158	87	87	344	3	8	8	5	5	55	760
Alphaproteobacteria	Sphingomonadales	Sphingomonadaceae	*Sphingobium*	218	156	36	17	70	108	1	12	95	43	756
Alphaproteobacteria	Rhodospirillales	Acetobacteraceae	*Roseococcus*	339	154	41	182	18	0	0	1	1	2	738

Clostridia	Clostridiales	Gracilibacteraceae	*Lutispora*	175	110	63	20	0	0	0	0	4	300	672
Negativicutes	Selenomonadales	Veillonellaceae	*Sporomusa*	25	21	119	12	0	0	0	0	41	441	659
Actinobacteria	Frankiales	Cryptosporangiaceae	*Fodinicola*	352	266	4	27	0	0	0	0	0	0	649
Gammaproteobacteria	Xanthomonadales	Xanthomonadaceae	*Stenotrophomonas*	149	78	315	55	1	4	0	1	0	1	604
Cytophagia	Cytophagales	Cytophagaceae	*Cytophaga*	78	36	129	52	0	0	0	33	270	0	598
Actinobacteria	Micrococcales	Sanguibacteraceae	*Sanguibacter*	230	177	15	134	6	0	0	2	23	0	587
Betaproteobacteria	Burkholderiales	Comamonadaceae	*Polaromonas*	189	145	137	95	1	2	0	3	0	8	580
Betaproteobacteria	Burkholderiales	Comamonadaceae	*Comamonas*	358	183	9	0	3	3	0	0	0	16	572
Alphaproteobacteria	Rhizobiales	Phyllobacteriaceae	*Mesorhizobium*	15	14	46	86	172	16	20	78	24	75	546
Actinobacteria	Propionibacteriales	Nocardioidaceae	*Aeromicrobium*	340	190	0	9	0	0	0	0	0	0	539
Gammaproteobacteria	Legionellales	Legionellaceae	*Legionella*	9	1	164	0	26	0	0	235	76	0	511
Flavobacteriia	Flavobacteriales	Cryomorphaceae	*Fluviicola*	2	1	8	3	0	0	0	0	0	460	474
Bacilli	Bacillales	Paenibacillaceae	*Paenibacillus*	95	68	98	0	5	7	3	1	79	115	471
Clostridia	Clostridiales	Clostridiales Family XI	*Sedimentibacter*	43	24	45	7	0	0	0	0	0	321	440
Cytophagia	Cytophagales	Cytophagaceae	*Dyadobacter*	176	75	133	8	0	0	1	0	4	40	437
Flavobacteriia	Flavobacteriales	Flavobacteriaceae	*Epilithonimonas*	6	10	74	0	0	96	63	5	33	145	432
Deltaproteobacteria	Myxococcales	Polyangiaceae	*Sorangium*	161	134	9	73	0	3	0	1	47	0	428
Sphingobacteriia	Sphingobacteriales	Chitinophagaceae	*Taibaiella*	163	79	28	21	0	0	50	2	46	38	427
Clostridia	Clostridiales	Lachnospiraceae	*Lachnoclostridium*	14	9	5	358	0	0	6	5	0	26	423
Alphaproteobacteria	Rhodospirillales	Rhodospirillaceae	*Skermanella*	0	0	6	0	0	0	1	5	398	0	410
Alphaproteobacteria	Rhizobiales	Rhizobiales IS	*Bauldia*	179	161	52	10	0	0	0	0	1	0	403
Acidimicrobiia	Acidimicrobiales	Acidimicrobiales	*Microthrix*	33	25	103	211	3	0	1	0	15	0	391
Opitutae	Opitutales	Opitutaceae	*Opitutus*	67	47	93	13	36	0	8	0	115	2	381
Alphaproteobacteria	Rhodospirillales	Acetobacteraceae	*Roseomonas*	17	28	98	218	8	0	0	0	0	2	371
Alphaproteobacteria	Rhodospirillales	Rhodospirillaceae	*Inquilinus*	10	0	3	0	288	0	0	2	67	0	370
Alphaproteobacteria	Rhizobiales	Bradyrhizobiaceae	*Rhodoblastus*	8	7	0	40	0	0	2	7	65	240	369
Betaproteobacteria	Neisseriales	Neisseriaceae	*Deefgea*	45	50	213	26	0	0	0	0	18	0	352
Gammaproteobacteria	Thiotrichales	Thiotrichales	*Endoecteinascidia*	0	0	0	0	0	0	0	0	340	0	340
Betaproteobacteria	Burkholderiales	Oxalobacteraceae	*Herminiimonas*	1	0	1	8	290	0	0	20	7	0	327
Sphingobacteriia	Sphingobacteriales	Chitinophagaceae	*Sediminibacterium*	0	0	0	0	22	0	48	29	221	0	320
Betaproteobacteria	Neisseriales	Neisseriaceae	*Paludibacterium*	0	0	0	0	0	0	0	158	156	0	314
Spartobacteria	Chthoniobacterales	Chthoniobacteraceae	*Chthoniobacter*	3	4	72	234	0	0	0	0	0	0	313
Alphaproteobacteria	Rhizobiales	Bradyrhizobiaceae	*Nitrobacter*	83	46	36	4	27	0	10	6	36	51	299
Clostridia	Clostridiales	Ruminococcaceae	*Ruminiclostridium*	137	19	1	0	0	31	0	76	14	3	281
Actinobacteria	Micrococcales	Microbacteriaceae	*Leucobacter*	70	45	17	107	10	0	2	2	0	0	253
Bacilli	Lactobacillales	Enterococcaceae	*Enterococcus*	16	3	0	0	0	110	0	5	115	0	249

Gammaproteobacteria	Enterobacteriales	Enterobacteriaceae	*Pantoea*	0	0	0	0	0	236	4	0	1	1	242
Verrucomicrobiae	Verrucomicrobiales	Verrucomicrobiaceae	*Brevifollis*	10	2	220	1	0	0	0	0	0	0	233
Cytophagia	Cytophagales	Cytophagaceae	*Emticicia*	54	21	0	148	2	0	0	0	0	0	225
Betaproteobacteria	Burkholderiales	Comamonadaceae	*Simplicispira*	1	5	45	4	9	64	31	13	2	40	214
Betaproteobacteria	Methylophilales	Methylophilaceae	*OM43*	60	31	73	3	0	0	0	0	0	40	207
Gammaproteobacteria	Xanthomonadales	Xanthomonadaceae	*Luteimonas*	99	72	3	29	0	0	0	0	0	0	203
Sphingobacteriia	Sphingobacteriales	Chitinophagaceae	*Chitinophaga*	1	2	22	0	1	0	1	0	8	166	201
Gammaproteobacteria	Pseudomonadales	Moraxellaceae	*Acinetobacter*	36	41	4	1	104	3	1	1	7	0	198
Gammaproteobacteria	Pseudomonadales	Moraxellaceae	*Perlucidibaca*	0	1	52	1	15	0	0	89	15	21	194
Cytophagia	Cytophagales	Cytophagaceae	*Persicitalea*	120	71	0	0	0	0	0	0	0	0	191
Betaproteobacteria	Burkholderiales	Burkholderiaceae	*Ralstonia*	18	5	2	3	116	8	3	13	14	7	189
Alphaproteobacteria	Caulobacterales	Hyphomonadaceae	*Hirschia*	11	22	11	140	0	0	0	0	3	0	187
Clostridia	Clostridiales	Lachnospiraceae	*Mobilitalea*	16	34	27	4	0	0	0	0	0	104	185
Betaproteobacteria	Burkholderiales	Burkholderiaceae	*Limnobacter*	0	0	1	0	81	0	4	25	66	8	185
Alphaproteobacteria	Rhodospirillales	Rhodospirillales IS	*Reyranella*	10	2	137	5	10	0	0	0	11	0	175
Sphingobacteriia	Sphingobacteriales	Sphingobacteriaceae	*Sphingobacterium*	80	38	3	4	0	0	0	0	0	28	153
Betaproteobacteria	Neisseriales	Neisseriaceae	*Chromobacterium*	0	0	0	0	0	3	98	17	10	21	149
Clostridia	Clostridiales	Ruminococcaceae	*Anaerobacterium*	2	3	143	0	0	0	0	0	0	0	148
Alphaproteobacteria	Rhizobiales	Bradyrhizobiaceae	*Bradyrhizobium*	21	15	2	1	54	1	6	10	9	27	146
Alphaproteobacteria	Rhizobiales	Methylobacteriaceae	*Methylobacterium*	21	4	5	2	99	8	0	3	0	0	142
Gammaproteobacteria	Xanthomonadales	Xanthomonadaceae	*Arenimonas*	0	0	5	136	0	0	0	0	0	0	141
Alphaproteobacteria	Rhodospirillales	Acetobacteraceae	*Acidisoma*	0	0	0	0	0	0	19	117	2	0	138
Alphaproteobacteria	Rickettsiales	Rickettsiales	*Captivus*	0	0	0	0	51	0	0	0	84	0	135
Alphaproteobacteria	Rickettsiales	Rickettsiales	*Hepatincola*	12	7	29	4	0	5	34	24	0	17	132
Bacteroidia	Bacteroidales	Porphyromonadaceae	*Dysgonomonas*	25	11	21	4	0	1	2	2	16	46	128
Sphingobacteriia	Sphingobacteriales	Chitinophagaceae	*Heliimonas*	0	0	0	0	60	0	54	6	6	0	126
Betaproteobacteria	Burkholderiales	Oxalobacteraceae	*Herbaspirillum*	1	0	2	0	119	0	0	0	2	0	124
Spartobacteria	Chthoniobacterales	Chthoniobacterales IS	*Terrimicrobium*	5	10	105	2	0	0	0	0	0	0	122
Alphaproteobacteria	Sphingomonadales	Erythrobacteraceae	*Erythrobacter*	0	0	0	0	0	0	3	110	1	0	114
Bacilli	Bacillales	Paenibacillaceae	*Cohnella*	51	24	5	2	0	0	0	0	0	28	110
Deltaproteobacteria	Myxococcales	Sandaracinaceae	*Sandaracinus*	18	24	0	67	0	1	0	0	0	0	110
Acidimicrobiia	Acidimicrobiales	Acidimicrobiaceae	*CL500-29 Marine group*	9	3	0	89	6	0	0	0	0	0	107
Cytophagia	Cytophagales	Cytophagaceae	*Spirosoma*	0	0	7	0	13	0	0	0	77	9	106
Deltaproteobacteria	Oligoflexales	Oligoflexaceae	*Oligoflexus*	56	47	0	0	0	0	0	0	0	0	103
Solibacteres	Solibacterales	Solibacteraceae	*Bryobacter*	0	1	2	95	0	0	0	0	3	0	101
Sphingobacteriia	Sphingobacteriales	Chitinophagaceae	*Dinghuibacter*	0	0	0	0	82	0	0	15	2	0	99

Clostridia	Clostridiales	Ruminococcaceae	*Caproiciproducens*	4	2	0	0	0	0	25	0	5	60	96
Betaproteobacteria	Burkholderiales	Burkholderiaceae	*Pandoraea*	0	0	0	0	26	1	1	67	0	1	96
Sphingobacteriia	Sphingobacteriales	Chitinophagaceae	*Filimonas*	2	0	6	1	0	0	0	0	78	7	94
Betaproteobacteria	Burkholderiales	Alcaligenaceae	*Achromobacter*	4	6	14	5	5	9	0	18	26	6	93
Alphaproteobacteria	Rhodospirillales	Rhodospirillaceae	*Phaeospirillum*	0	0	0	0	0	0	0	0	85	0	85
Actinobacteria	Micrococcales	Cellulomonadaceae	*Actinotalea*	10	3	3	26	4	0	3	20	8	1	78
Deltaproteobacteria	Bdellovibrionales	Bacteriovoracaceae	*Peredibacter*	3	1	43	2	14	2	0	0	0	12	77
Sphingobacteriia	Sphingobacteriales	Chitinophagaceae	*Terrimonas*	23	3	6	42	0	0	0	2	0	0	76
Actinobacteria	Frankiales	Frankiaceae	*Jatrophihabitans*	2	0	2	1	39	0	6	3	21	0	74
Betaproteobacteria	Burkholderiales	Comamonadaceae	*Sphaerotilus*	0	0	0	0	31	1	7	23	9	2	73
Gammaproteobacteria	Chromatiales	Chromatiaceae	*Rheinheimera*	17	6	16	23	4	0	0	0	0	0	66
Alphaproteobacteria	Rhodospirillales	Acetobacteraceae	*Rhodovastum*	0	0	6	0	23	0	2	13	21	0	65
Alphaproteobacteria	Sphingomonadales	Sphingomonadaceae	*Zymomonas*	12	0	0	1	10	3	23	4	2	7	62
Betaproteobacteria	Burkholderiales	Comamonadaceae	*Ideonella*	2	2	0	58	0	0	0	0	0	0	62
Gammaproteobacteria	Enterobacteriales	Enterobacteriaceae	*Citrobacter*	0	1	0	0	0	0	0	1	0	59	61
Actinobacteria	Frankiales	Nakamurellaceae	*Nakamurella*	7	1	24	27	1	0	0	0	0	0	60
Actinobacteria	Micrococcales	Microbacteriaceae	*Frigoribacterium*	20	17	2	19	0	0	0	0	1	0	59
Alphaproteobacteria	Rhizobiales	Hyphomicrobiaceae	*Blastochloris*	31	28	0	0	0	0	0	0	0	0	59
Actinobacteria	Micrococcales	Microbacteriaceae	*Microbacterium*	10	3	0	7	8	1	7	19	3	0	58
Alphaproteobacteria	Rhizobiales	Xanthobacteraceae	*Xanthobacter*	1	1	55	1	0	0	0	0	0	0	58
Betaproteobacteria	Rhodocyclales	Rhodocyclaceae	*Uliginosibacterium*	21	29	0	2	0	0	0	0	5	0	57
Actinobacteria	Micrococcales	Cellulomonadaceae	*Paraoerskovia*	2	5	0	47	0	0	0	0	0	0	54
Alphaproteobacteria	Rhizobiales	Beijerinckiaceae	*Methylocella*	0	0	0	0	1	0	3	30	18	1	53
Alphaproteobacteria	Rhizobiales	Phyllobacteriaceae	*Phyllobacterium*	4	1	40	8	0	0	0	0	0	0	53
Betaproteobacteria	Burkholderiales	Oxalobacteraceae	*Noviherbaspirillum*	5	9	5	0	1	0	2	30	1	0	53
Betaproteobacteria	Burkholderiales	Comamonadaceae	*Pseudorhodoferax*	7	4	23	5	0	1	0	0	0	12	52
Bacilli	Lactobacillales	Carnobacteriaceae	*Isobaculum*	0	0	0	0	0	0	0	0	52	0	52
Betaproteobacteria	Burkholderiales	Comamonadaceae	*Mitsuaria*	1	1	0	0	49	0	0	0	0	0	51
Betaproteobacteria	Burkholderiales	Oxalobacteraceae	*Oxalobacter*	0	0	0	0	0	0	0	51	0	0	51
Clostridia	Clostridiales	Lachnospiraceae	*Lachnoclostridium*	1	0	3	0	0	1	2	0	8	35	50
Actinobacteria	Micrococcales	Microbacteriaceae	*Klugiella*	3	4	1	34	1	0	1	2	3	1	50
Betaproteobacteria	Neisseriales	Neisseriaceae	*Pseudogulbenkiania*	0	0	0	0	0	0	0	10	39	0	49
Cytophagia	Cytophagales	Cytophagaceae	*Leadbetterella*	19	5	24	0	0	0	0	0	0	0	48
Betaproteobacteria	Neisseriales	Neisseriaceae	*Vitreoscilla*	17	3	6	1	0	0	14	0	7	0	48
Bacilli	Lactobacillales	Carnobacteriaceae	*Carnobacterium*	19	22	1	0	0	4	0	0	1	0	47
Gammaproteobacteria	Orbales	Orbaceae	*Gilliamella*	0	0	0	0	0	0	0	0	46	0	46

Gammaproteobacteria	Xanthomonadales	Xanthomonadaceae	*Pseudoxanthomonas*	4	0	8	0	7	0	3	9	15	0	46
Verrucomicrobiae	Verrucomicrobiales	Verrucomicrobiaceae	*Haloferula*	0	0	0	0	0	0	0	0	0	45	45
Planctomycetacia	Planctomycetales	Planctomycetaceae	*Isosphaera*	0	0	5	0	2	0	0	1	36	0	44
Gammaproteobacteria	Xanthomonadales	Xanthomonadales IS	*Acidibacter*	0	0	0	0	43	0	0	0	1	0	44
Acidobacteria	Acidobacteriales	Acidobacteriaceae	*Acidobacterium*	0	0	0	0	43	0	0	0	0	0	43
Alphaproteobacteria	Rhizobiales	Xanthobacteraceae	*Pseudolabrys*	5	1	1	0	0	0	0	0	19	16	42
Gammaproteobacteria	Enterobacteriales	Enterobacteriaceae	*Yersinia*	0	0	0	0	16	11	9	6	0	0	42
Alphaproteobacteria	Rhodospirillales	Acetobacteraceae	*Acidisphaera*	0	0	0	0	8	0	0	2	31	0	41
Gammaproteobacteria	Enterobacteriales	Enterobacteriaceae	*Raoultella*	0	0	0	0	0	5	0	0	1	34	40
Betaproteobacteria	Neisseriales	Neisseriaceae	*Chitiniphilus*	0	0	0	0	0	6	0	9	5	19	39
Actinobacteria	Corynebacteriales	Nocardiaceae	*Nocardia*	14	14	10	0	0	0	0	0	0	0	38
Alphaproteobacteria	Rhodospirillales	Rhodospirillaceae	*Telmatospirillum*	0	0	0	0	0	0	0	0	37	0	37
Blastocatellia	Blastocatellales	Blastocatellaceae	*Blastocatella*	30	4	0	0	0	0	0	0	2	0	36
Alphaproteobacteria	Rhizobiales	Rhizobiales	*Phreatobacter*	0	0	2	0	2	0	0	0	2	29	35
Alphaproteobacteria	Rhizobiales	Roseiarcaceae	*Roseiarcus*	0	0	0	0	11	0	0	11	10	2	34
Betaproteobacteria	Neisseriales	Neisseriaceae	*Microvirgula*	2	0	9	0	0	0	0	0	23	0	34
Alphaproteobacteria	Rhizobiales	Brucellaceae	*Ochrobactrum*	0	0	2	0	0	3	0	0	2	25	32
Betaproteobacteria	Rhodocyclales	Rhodocyclaceae	*Georgfuchsia*	2	5	0	0	25	0	0	0	0	0	32
Verrucomicrobiae	Verrucomicrobiales	Verrucomicrobiaceae	*Luteolibacter*	2	4	10	2	0	0	0	0	6	7	31
Alphaproteobacteria	Rhizobiales	Xanthobacteraceae	*Variibacter*	8	2	8	8	5	0	0	0	0	0	31
Gammaproteobacteria	Enterobacteriales	Enterobacteriaceae	*Pectobacterium*	0	0	0	0	0	2	0	23	0	4	29
Sphingobacteriia	Sphingobacteriales	Sphingobacteriaceae	*Arcticibacter*	9	1	0	15	0	0	3	0	1	0	29
Alphaproteobacteria	Sphingomonadales	Sphingomonadaceae	*Sandaracinobacter*	0	0	11	0	0	6	2	4	6	0	29
Alphaproteobacteria	Rhizobiales	Xanthobacteraceae	*Labrys*	0	0	1	2	0	0	0	7	18	0	28
Betaproteobacteria	Burkholderiales	Comamonadaceae	*Brachymonas*	9	13	0	6	0	0	0	0	0	0	28
Betaproteobacteria	Burkholderiales	Comamonadaceae	*Paucibacter*	1	2	0	5	20	0	0	0	0	0	28
Actinobacteria	Propionibacteriales	Nocardioidaceae	*Nocardioides*	0	4	0	0	16	0	0	0	7	0	27
Clostridia	Clostridiales	Peptococcaceae	*Desulfosporosinus*	8	3	16	0	0	0	0	0	0	0	27
Clostridia	Clostridiales	Ruminococcaceae	*Anaerotruncus*	0	0	0	0	27	0	0	0	0	0	27
Gammaproteobacteria	Legionellales	Coxiellaceae	*Coxiella*	0	0	2	0	4	0	0	0	21	0	27
Gammaproteobacteria	Enterobacteriales	Enterobacteriaceae	*Escherichia-Shigella*	0	0	2	0	0	11	1	2	1	9	26
Alphaproteobacteria	Rhizobiales	Rhizobiaceae	*Shinella*	7	2	4	8	3	0	0	1	0	1	26
Acidobacteria	Acidobacteriales	Acidobacteriaceae	*Acidicapsa*	0	0	0	0	25	0	0	0	0	0	25
Sphingobacteriia	Sphingobacteriales	Chitinophagaceae	*Flavitalea*	9	5	11	0	0	0	0	0	0	0	25
Sphingobacteriia	Sphingobacteriales	Saprospiraceae	*Portibacter*	9	16	0	0	0	0	0	0	0	0	25
Alphaproteobacteria	Rhizobiales	Beijerinckiaceae	*Chelatococcus*	8	6	6	5	0	0	0	0	0	0	25

Alphaproteobacteria	Rickettsiales	Rickettsiaceae	*Trichorickettsia*	0	0	2	0	4	0	0	0	19	0	25
Gammaproteobacteria	Oceanospirillales	Oceanospirillaceae	*Pseudohongiella*	13	3	0	7	0	0	0	0	2	0	25
Gammaproteobacteria	Thiotrichales	Thiotrichaceae	*Thiothrix*	17	6	0	0	2	0	0	0	0	0	25
Spirochaetes	Spirochaetales	Spirochaetaceae	*Spirochaeta*	0	0	2	0	0	0	0	0	0	22	24
Betaproteobacteria	Burkholderiales	Comamonadaceae	*Curvibacter*	0	1	6	0	0	0	0	1	8	8	24
Phycisphaerae	Phycisphaerales	Phycisphaeraceae	*SM1A02*	11	12	0	1	0	0	0	0	0	0	24
Alphaproteobacteria	Rickettsiales	Rickettsiaceae	*Rickettsia*	0	0	1	0	9	1	0	0	12	0	23
Acidobacteria	Acidobacteriales	Acidobacteriaceae	*Bryocella*	0	0	0	0	2	0	1	15	4	0	22
Gammaproteobacteria	Enterobacteriales	Enterobacteriaceae	*Pseudocitrobacter*	0	0	0	0	0	0	0	0	0	21	21
Alphaproteobacteria	Rhizobiales	Hyphomicrobiaceae	*Hyphomicrobium*	10	2	2	7	0	0	0	0	0	0	21
Alphaproteobacteria	Rhodospirillales	Acetobacteraceae	*Acidiphilium*	0	0	0	0	10	0	9	1	1	0	21
Betaproteobacteria	Burkholderiales	Comamonadaceae	*Limnohabitans*	5	6	1	1	0	0	0	0	8	0	21
Betaproteobacteria	Rhodocyclales	Rhodocyclaceae	*Thauera*	5	2	8	0	0	0	0	0	6	0	21
Deltaproteobacteria	Myxococcales	Haliangiaceae	*Haliangium*	0	4	0	0	5	0	0	0	12	0	21
Actinobacteria	Micrococcales	Micrococcaceae	*Paeniglutamicibacter*	10	10	0	0	0	0	0	0	0	0	20
Bacilli	Bacillales	Listeriaceae	*Listeria*	17	1	0	1	0	0	0	0	0	0	19
Betaproteobacteria	Burkholderiales	Alcaligenaceae	*Verticia*	7	4	0	8	0	0	0	0	0	0	19
Betaproteobacteria	Burkholderiales	Oxalobacteraceae	*Aquaspirillum*	0	0	0	0	19	0	0	0	0	0	19
Gammaproteobacteria	Enterobacteriales	Enterobacteriaceae	*Buttiauxella*	0	0	0	0	2	6	3	0	1	6	18
Actinobacteria	Micrococcales	Microbacteriaceae	*Amnibacterium*	0	0	0	0	17	0	0	0	1	0	18
Actinobacteria	Pseudonocardiales	Pseudonocardiaceae	*Actinomycetospora*	1	1	1	0	10	0	0	0	5	0	18
Alphaproteobacteria	Rhizobiales	Phyllobacteriaceae	*Aminobacter*	0	0	8	9	0	0	0	0	1	0	18
Betaproteobacteria	Burkholderiales	Comamonadaceae	*Ottowia*	8	7	0	2	1	0	0	0	0	0	18
Gammaproteobacteria	Xanthomonadales	Xanthomonadaceae	*Chiayiivirga*	0	1	0	17	0	0	0	0	0	0	18
Betaproteobacteria	Burkholderiales	Oxalobacteraceae	*Paucimonas*	0	0	5	0	0	0	12	0	0	0	17
Planctomycetacia	Planctomycetales	Planctomycetaceae	*Singulisphaera*	0	0	6	2	0	0	0	0	8	0	16
Alphaproteobacteria	Rickettsiales	Rickettsiales IS	*Odyssella*	0	0	0	0	16	0	0	0	0	0	16
Gammaproteobacteria	Xanthomonadales	Xanthomonadaceae	*Thermomonas*	8	8	0	0	0	0	0	0	0	0	16
Chthonomonadetes	Chthonomonadales	Chthonomonadaceae	*Chthonomonas*	0	0	0	0	15	0	0	0	0	0	15
Alphaproteobacteria	Sphingomonadales	Erythrobacteraceae	*Altererythrobacter*	5	2	1	5	0	0	0	2	0	0	15
Alphaproteobacteria	Sphingomonadales	Sphingomonadaceae	*Sphingorhabdus*	0	0	0	0	0	0	0	4	11	0	15
Betaproteobacteria	Neisseriales	Neisseriaceae	*Amantichitinum*	0	0	0	0	0	3	0	3	5	3	14
Alphaproteobacteria	Rhizobiales	Xanthobacteraceae	*Starkeya*	1	0	9	3	0	0	0	0	0	1	14
Actinobacteria	Streptomycetales	Streptomycetaceae	*E1B-B3-114*	0	5	5	1	0	3	0	0	0	0	14
Clostridia	Clostridiales	Lachnospiraceae	*Lachnospiraceae UCG-007*	0	1	11	2	0	0	0	0	0	0	14
Alphaproteobacteria	Rhodobacterales	Rhodobacteraceae	*Rhodobacter*	3	7	2	0	0	0	0	0	2	0	14

Betaproteobacteria	Burkholderiales	Comamonadaceae	*Leptothrix*	2	2	0	0	10	0	0	0	0	0	14
Gammaproteobacteria	Legionellales	Coxiellaceae	*Aquicella*	0	0	0	0	12	0	0	0	2	0	14
Gammaproteobacteria	Xanthomonadales	Xanthomonadaceae	*Ignatzschineria*	3	0	4	0	0	2	0	0	2	2	13
Deltaproteobacteria	Myxococcales	Phaselicystidaceae	*Phaselicystis*	5	0	0	0	0	0	2	0	6	0	13
Gammaproteobacteria	Enterobacteriales	Enterobacteriaceae	*Erwinia*	0	1	5	0	0	1	0	0	2	3	12
Cytophagia	Cytophagales	Cytophagaceae	*Hymenobacter*	0	0	0	0	12	0	0	0	0	0	12
Flavobacteriia	Flavobacteriales	Flavobacteriaceae	*Cloacibacterium*	1	2	0	0	7	1	0	0	1	0	12
Clostridia	Clostridiales	Ruminococcaceae	*Ruminiclostridium*	0	0	9	0	0	0	0	3	0	0	12
Alphaproteobacteria	Rhizobiales	Hyphomicrobiaceae	*Methylorhabdus*	5	6	1	0	0	0	0	0	0	0	12
Actinobacteria	Micrococcales	Microbacteriaceae	*Curtobacterium*	0	0	2	0	4	0	0	2	3	0	11
Betaproteobacteria	Burkholderiales	Alcaligenaceae	*Castellaniella*	0	0	0	0	11	0	0	0	0	0	11
Betaproteobacteria	Burkholderiales	Comamonadaceae	*Azohydromonas*	0	0	0	11	0	0	0	0	0	0	11
Actinobacteria	Corynebacteriales	Nocardiaceae	*Williamsia*	2	0	0	8	0	0	0	0	0	0	10
Betaproteobacteria	Burkholderiales	Burkholderiaceae	*Polynucleobacter*	1	0	7	0	2	0	0	0	0	0	10
Clostridia	Clostridiales	Peptostreptococcaceae	*Romboutsia*	2	5	0	0	1	0	0	0	0	1	9
Actinobacteria	Micrococcales	Micrococcaceae	*Arthrobacter*	9	0	0	0	0	0	0	0	0	0	9
Sphingobacteriia	Sphingobacteriales	Chitinophagaceae	*Flavihumibacter*	0	0	5	4	0	0	0	0	0	0	9
Alphaproteobacteria	Rhodobacterales	Rhodobacteraceae	*Amaricoccus*	0	0	0	0	9	0	0	0	0	0	9
Alphaproteobacteria	Rhodospirillales	Acetobacteraceae	*Craurococcus*	0	0	7	1	0	0	0	1	0	0	9
Alphaproteobacteria	Rhodospirillales	Rhodospirillaceae	*Nitrospirillum*	0	0	0	0	0	0	0	0	9	0	9
Betaproteobacteria	Nitrosomonadales	Gallionellaceae	*Sideroxydans*	0	0	0	0	6	0	0	3	0	0	9
Gammaproteobacteria	Enterobacteriales	Enterobacteriaceae	*Klebsiella*	0	0	3	0	0	3	0	0	1	1	8
Actinobacteria	Frankiales	Sporichthyaceae	*Sporichthya*	3	4	0	1	0	0	0	0	0	0	8
Actinobacteria	Propionibacteriales	Propionibacteriaceae	*uncultured*	7	0	0	1	0	0	0	0	0	0	8
Betaproteobacteria	Burkholderiales	Comamonadaceae	*Albidiferax*	0	0	0	0	3	0	4	0	1	0	8
Gammaproteobacteria	Enterobacteriales	Enterobacteriaceae	*Blochmannia*	0	0	0	0	0	0	8	0	0	0	8
Gammaproteobacteria	Enterobacteriales	Enterobacteriaceae	*Enterobacter*	0	0	0	0	0	6	0	0	2	0	8
Gammaproteobacteria	Xanthomonadales	Xanthomonadaceae	*Luteibacter*	0	0	0	0	7	1	0	0	0	0	8
Gammaproteobacteria	Enterobacteriales	Enterobacteriaceae	*Lelliottia*	0	0	0	0	0	0	0	0	0	7	7
Flavobacteriia	Flavobacteriales	Flavobacteriaceae	*Bergeyella*	0	0	0	0	0	0	3	0	1	3	7
Acidobacteria	Acidobacteriales	Acidobacteriaceae	*Edaphobacter*	0	0	0	0	7	0	0	0	0	0	7
Actinobacteria	Micrococcales	Microbacteriaceae	*Pseudoclavibacter*	2	1	1	2	0	0	0	1	0	0	7
Alphaproteobacteria	Rhizobiales	Xanthobacteraceae	*Pseudoxanthobacter*	2	0	5	0	0	0	0	0	0	0	7
Betaproteobacteria	Rhodocyclales	Rhodocyclaceae	*Azoarcus*	0	0	0	0	7	0	0	0	0	0	7
Deltaproteobacteria	Myxococcales	Polyangiaceae	*Labilithrix*	0	0	0	7	0	0	0	0	0	0	7
Gammaproteobacteria	Acidiferrobacterales	Acidiferrobacteraceae	*Sulfurifustis*	0	0	0	0	7	0	0	0	0	0	7

Gammaproteobacteria	Xanthomonadales	Xanthomonadaceae	*Lysobacter*	1	1	3	2	0	0	0	0	0	0	7
Synergistia	Synergistales	Synergistaceae	*Aminivibrio*	0	0	5	0	1	0	0	0	1	0	7
Clostridia	Clostridiales	Ruminococcaceae	*Ruminiclostridium*	0	0	5	0	0	0	0	0	0	1	6
Actinobacteria	Kineosporiales	Kineosporiaceae	*Quadrisphaera*	0	0	0	1	2	0	0	2	1	0	6
Actinobacteria	Micrococcales	Microbacteriaceae	*Conyzicola*	1	0	0	5	0	0	0	0	0	0	6
Sphingobacteriia	Sphingobacteriales	Chitinophagaceae	*Niabella*	3	0	0	3	0	0	0	0	0	0	6
Alphaproteobacteria	Rhizobiales	Hyphomicrobiaceae	*Rhodomicrobium*	0	0	3	1	2	0	0	0	0	0	6
Alphaproteobacteria	Rickettsiales	Rickettsiaceae	*Arcanobacter*	0	0	0	0	6	0	0	0	0	0	6
Betaproteobacteria	Neisseriales	Neisseriaceae	*Leeia*	5	0	1	0	0	0	0	0	0	0	6
Gammaproteobacteria	Xanthomonadales	Nevskiaceae	*Nevskia*	0	0	0	0	0	0	2	4	0	0	6
Gammaproteobacteria	Xanthomonadales	Xanthomonadaceae	*Dyella*	0	0	0	0	0	0	0	3	3	0	6
Gammaproteobacteria	Aeromonadales	Aeromonadaceae	*Aeromonas*	0	0	0	0	0	0	5	1	0	0	6
Betaproteobacteria	Burkholderiales	Oxalobacteraceae	*Oxalicibacterium*	0	0	0	0	0	0	0	0	4	1	5
Actinobacteria	Frankiales	Geodermatophilaceae	*Geodermatophilus*	0	2	0	3	0	0	0	0	0	0	5
Actinobacteria	Micrococcales	Intrasporangiaceae	*Oryzihumus*	0	0	0	0	5	0	0	0	0	0	5
Chloroflexia	Chloroflexales	Oscillochloridaceae	*Oscillochloris*	0	0	0	0	5	0	0	0	0	0	5
Clostridia	Clostridiales	Eubacteriaceae	*Alkalibacter*	4	1	0	0	0	0	0	0	0	0	5
Clostridia	Clostridiales	Lachnospiraceae	*Epulopiscium*	0	0	0	5	0	0	0	0	0	0	5
Clostridia	Clostridiales	Lachnospiraceae	*Tyzzerella*	0	0	3	0	0	0	0	1	1	0	5
Alphaproteobacteria	Rhizobiales	Rhizobiales IS	*Agaricicola*	0	0	0	5	0	0	0	0	0	0	5
Alphaproteobacteria	Rhodobacterales	Rhodobacteraceae	*Falsirhodobacter*	0	0	0	5	0	0	0	0	0	0	5
Alphaproteobacteria	Rhodospirillales	Rhodospirillales	*Alysiosphaera*	2	0	3	0	0	0	0	0	0	0	5
Betaproteobacteria	Burkholderiales	Comamonadaceae	*Alicycliphilus*	0	3	1	1	0	0	0	0	0	0	5
Betaproteobacteria	Burkholderiales	Comamonadaceae	*Symbiobacter*	0	0	0	1	0	0	0	1	3	0	5
Betaproteobacteria	Burkholderiales	Comamonadaceae	*Rhodoferax*	1	0	0	1	1	0	0	0	2	0	5
Betaproteobacteria	Neisseriales	Neisseriaceae	*Vogesella*	0	0	0	0	0	0	0	0	5	0	5
Gammaproteobacteria	Xanthomonadales	Xanthomonadaceae	*Dokdonella*	0	0	0	0	0	0	0	3	2	0	5
Spartobacteria	Chthoniobacterales	Xiphinematobacteraceae	*Xiphinematobacter*	3	0	2	0	0	0	0	0	0	0	5
Alphaproteobacteria	Sphingomonadales	Sphingomonadaceae	*Rhizorhapis*	0	0	1	0	0	0	0	0	0	3	4
Actinobacteria	Micrococcales	Promicromonosporaceae	*Cellulosimicrobium*	0	1	0	2	1	0	0	0	0	0	4
Actinobacteria	Streptomycetales	Streptomycetaceae	*Streptomyces*	0	0	0	4	0	0	0	0	0	0	4
Cytophagia	Cytophagales	Cytophagaceae	*Larkinella*	1	1	0	2	0	0	0	0	0	0	4
Sphingobacteriia	Sphingobacteriales	Sphingobacteriaceae	*Pedobacterms03_R*	0	0	0	0	0	0	4	0	0	0	4
Chlamydiae	Chlamydiales	Parachlamydiaceae	*Neochlamydia*	0	0	0	0	4	0	0	0	0	0	4
Cyanobacteria	SubsectionI	FamilyI	*Prochlorococcus*	0	0	0	0	0	0	1	1	2	0	4
Planctomycetacia	Planctomycetales	Planctomycetaceae	*Pirellula*	0	2	0	2	0	0	0	0	0	0	4

Alphaproteobacteria	Rhizobiales	Methylocystaceae	*Methylosinus*	0	2	0	2	0	0	0	0	0	0	4
Alphaproteobacteria	Rhizobiales	Rhizobiaceae	*Ensifer*	0	0	2	2	0	0	0	0	0	0	4
Alphaproteobacteria	Rickettsiales	Rickettsiales IS	*Finniella*	0	0	0	0	0	0	2	2	0	0	4
Betaproteobacteria	Methylophilales	Methylophilaceae	*Methylophilus*	0	0	2	0	0	0	0	2	0	0	4
Actinobacteria	Catenulisporales	Actinospicaceae	*Actinospica*	0	0	0	0	3	0	0	0	0	0	3
Cytophagia	Cytophagales	Cytophagaceae	*Sporocytophaga*	0	0	3	0	0	0	0	0	0	0	3
Chlamydiae	Chlamydiales	Simkaniaceae	*Renichlamydia*	0	0	0	0	0	0	2	1	0	0	3
Clostridia	Clostridiales	Peptostreptococcaceae	*Peptoclostridium*	1	2	0	0	0	0	0	0	0	0	3
Alphaproteobacteria	Caulobacterales	Hyphomonadaceae	*Woodsholea*	0	0	0	3	0	0	0	0	0	0	3
Alphaproteobacteria	Rhizobiales	C2U	*Pseudoxanthobacter*	1	2	0	0	0	0	0	0	0	0	3
Alphaproteobacteria	Rhizobiales	Methylocystaceae	*Methylocystis*	0	0	3	0	0	0	0	0	0	0	3
Alphaproteobacteria	Rhodospirillales	Acetobacteraceae	*Humitalea*	0	0	0	3	0	0	0	0	0	0	3
Alphaproteobacteria	Rickettsiales	Anaplasmataceae	*Wolbachia*	0	0	0	0	0	3	0	0	0	0	3
Betaproteobacteria	Burkholderiales	Oxalobacteraceae	*Glaciimonas*	0	0	0	0	3	0	0	0	0	0	3
Deltaproteobacteria	Myxococcales	Polyangiaceae	*Aetherobacter*	0	0	0	0	3	0	0	0	0	0	3
Gammaproteobacteria	Thiotrichales	Thiotrichaceae	*Beggiatoa*	0	0	0	0	3	0	0	0	0	0	3
Alphaproteobacteria	Rhodobacterales	Rhodobacteraceae	*Roseivivax*	0	0	0	0	0	0	0	0	0	2	2
Betaproteobacteria	Burkholderiales	Comamonadaceae	*Giesbergeria*	0	0	0	0	0	0	0	0	0	2	2
Gammaproteobacteria	Enterobacteriales	Enterobacteriaceae	*Cronobacter*	0	0	0	0	0	0	0	0	0	2	2
Gammaproteobacteria	Enterobacteriales	Enterobacteriaceae	*Rosenbergiella*	0	0	0	0	0	0	0	0	0	2	2
Gammaproteobacteria	Enterobacteriales	Enterobacteriaceae	*Salmonella*	0	0	0	0	0	0	0	0	0	2	2
Alphaproteobacteria	Rhizobiales	Bradyrhizobiaceae	*Tardiphaga*	0	0	0	1	0	0	0	0	0	1	2
Acidimicrobiia	Acidimicrobiales	Acidimicrobiaceae	*Ilumatobacter*	0	1	0	1	0	0	0	0	0	0	2
Actinobacteria	Micrococcales	Microbacteriaceae	*Lysinimonas*	1	0	0	1	0	0	0	0	0	0	2
Actinobacteria	Micromonosporales	Micromonosporaceae	*Micromonospora*	0	0	0	0	2	0	0	0	0	0	2
Thermoleophilia	Solirubrobacterales	Solirubrobacteraceae	*Solirubrobacter*	0	0	0	2	0	0	0	0	0	0	2
Sphingobacteriia	Sphingobacteriales	Sphingobacteriaceae	*Nubsella*	0	0	0	0	0	2	0	0	0	0	2
Planctomycetacia	Planctomycetales	Planctomycetaceae	*Schlesneria*	0	0	0	0	2	0	0	0	0	0	2
Alphaproteobacteria	Rhizobiales	Phyllobacteriaceae	*Nitratireductor*	0	0	2	0	0	0	0	0	0	0	2
Alphaproteobacteria	Rhodospirillales	Acetobacteraceae	*Acidocella*	0	0	0	0	0	0	0	2	0	0	2
Alphaproteobacteria	Rhodospirillales	Acetobacteraceae	*Endobacter*	0	0	0	0	2	0	0	0	0	0	2
Alphaproteobacteria	Sphingomonadales	Sphingomonadaceae	*Sphingomonas WWII*	1	0	0	1	0	0	0	0	0	0	2
Betaproteobacteria	Burkholderiales	Burkholderiaceae	*Cupriavidus*	0	0	2	0	0	0	0	0	0	0	2
Betaproteobacteria	Hydrogenophilales	Hydrogenophilaceae	*Thiobacillus*	0	0	0	0	0	0	0	0	2	0	2
Betaproteobacteria	Rhodocyclales	Rhodocyclaceae	*Azovibrio*	0	1	1	0	0	0	0	0	0	0	2
Deltaproteobacteria	Myxococcales	Polyangiaceae	*Polyangium*	0	0	0	0	2	0	0	0	0	0	2

Gammaproteobacteria	Enterobacteriales	Enterobacteriaceae	*Sodalis*	0	0	0	0	0	2	0	0	0	0	2
Gammaproteobacteria	Thiotrichales	Piscirickettsiaceae	*Methylophaga*	1	1	0	0	0	0	0	0	0	0	2
