# Supplementary material for: Diverse microbial communities hosted by the model carnivorous pitcher plant Sarracenia purpurea: analysis of both bacterial and eukaryotic composition across distinct host plant populations
Source: PeerJ. 2019 Feb 18;7:e6392. doi: 10.7717/peerj.6392 (PMC6383556; doi:10.7717/peerj.6392)
Supplement: Table S2 — Details of Eukaryotic taxa including Genus-species details (where available), identified in samples from 18S rRNA sequence analysis. Taxa are grouped by higher taxonomic affinity, which was determined using databases Algaebase.org, WORMS (www.marinespecies.org/), encyclopedia of life (eol.org). Totals for major groups are listed and Cedarburg and Sapa totals are show in shaded columns. Abbreviations: unc –uncultured, unclass –unclassified, IS –incertae sedis. Blank cells mean no counts were identified. [file peerj-07-6392-s005.pdf]

Table S2. Details of Eukaryotic taxa including Genus-species details (where available), identified in samples from 18S rRNA sequence analysis. Taxa are grouped by higher taxonomic affinity, which was determined using databases Algaebase.org, WORMS (www.marinespecies.org/), encyclopedia of life (eol.org). Totals for major groups are listed and Cedarburg and Sapa totals are show in shaded columns. Abbreviations: unc – uncultured, unclass – unclassified, IS – incertae sedis. Blank cells mean no counts were identified.

			Median adjusted sequence number per sample													
Class	Family	Genus species	CB1p1	CB1p2	CB1p3	CB1p4	CB5p234	Cedarburg Totals	Sp3p2	Sp3p4	Sp3p5	Sp5p23	Sp9p234	Sapa Totals	Taxon totals	top 25 taxon rank
Ciliates (Alveolata)																
Colpodea	Colpodidae	*Colpoda_unc*						0	167					167	167	
	Cyrtolophosidida_IS	*Cyrtolophosidida_unc*				39		39	167				50	218	257	
	Cyrtolophosididae	*Cyrtolophosidida_unc*						0				39		39	39	
	Cyrtolophosididae	*Cyrtolophosis mucicola*						0			298			298	298	
	Exocolpodidae	*Exocolpoda augustini*		10	60		329	399						0	399	
	Grossglockneriidae	*Mykophagophrys terricola*	603	19	1219	63	958	2862					36	36	2898	19
	Grossglockneriidae	*Pseudoplatyophyra_unc*			37	145		182	258		16	506	31	810	992	
	Hausmanniellidae	*Hausmanniella discoidea*		72	0	73	283	428		46				46	474	
	Woodruffiidae	*Rostrophrya sp. MSD-2007*						0			48			48	48	
Nassophorea	Microthoracida	*Microthoracida_unc*						0		111	28			139	139	
	Colpodidae	*Colpoda steinii*	12		476	70		558					18	18	577	
	Colpodidae	*Colpodida_unc*						0	12021				838	12859	12859	12
	Leptopharynidae	*Leptopharynx costatus*	18	14		106		138	2042		157	208	146	2553	2690	20
Oligohymenophorea	Epistylidae	*Campanella umbellaria*					2250	2250						0	2250	23
	Frontoniidae	*Apofrontonia dohrni*						0		533	250	10		793	793	
	Orchitophryidae	*Orchitophryidae_env_samp*	1003	959	21911	91016	4723	119613	5479	42	344	1738	46441	54044	173657	2
	Loxocephalidae	*Cardiostomatella_unc*						0				37		37	37	
	Scuticociliatia_IS	*Scuticociliatia_unc*	22	15		161	52537	52735		66678	62242	1543	9	130472	183207	1
	Tetrahymenidae	*Tetrahymena sp. NI*						0	19084	96	17		12	19209	19209	10
	Uronematidae	*Homalogastra setosa*						0				8		8	8	
	Ancistridae	*Ancistrum_unc*						0			14			14	14	
Prostomatea	Cryptocaryonidae	*Cryptocaryon_unc*		8	15		50	74	6224	15169	24046	47350		92789	92863	5
	Prorodontidae	*Prorodon_unc*						0				34		34	34	
		**Total Ciliates**	**1658**	**1097**	**23718**	**91675**	**61129**	**179277**	**45442**	**82675**	**87460**	**51473**	**47581**	**314631**	**493908**	
Parasitic Apicomplexans																
Conoidasida	Actinocephalidae	*Paraschneideria metamorphosa*						0	329					329	329	
	Eimeriidae	*Eimeriidae (Elev-18S-1089)_unc*						0				19		19	19	
	Eimeriidae	*Eimeriidae_unc*						0				27		27	27	
Athropoda																
Arachnida - mites	Bdellidae	*Bdellodes sp. AP-2010*			18			18						0	18	
	Cymbaeremaeidae	*Cymbaeremaeus cymba*						0			196			196	196	
	Eniochthoniidae	*Eniochthonius sp. AD1066*						0				263		263	263	
	Epilohmanniidae	*Epilohmannia sp. 1 KD-2008*						0		14				14	14	
	Eupodidae	*Eupodidae sp. AMUENV025*						0		112	17		13	142	142	
	Haplozetidae	*Rostrozetes sp. 1 IS-2008*			227			227					43	43	270	
	Histiostomatidae	*Ovanoetus sp. AD678*	20		22			42	463			16	50	529	570	
	Liacaridae	*Adoristes ovatus*						0	55					55	55	
	Sarcoptiformes_Unclass	*Sarcoptiformes sp. JA-2011*						0				240		240	240	
	Phenopelopidae	*Eupelops hirtus*	16		35			51	471	32			1746	2250	2301	22
	Trhypochthoniidae	*Afronothrus sp. AP-2010*						0				215		215	215	
	Trhypochthoniidae	*Trhypochthonius silvestris europaeus*				2116		2116			2448			2448	4564	15
	Trombidiidae	*Trombidiinae sp. AMUENV055*						0					10	10	10	
Arachnida - spiders	Theridiidae	*Enoplognatha margarita*						0			566			566	566	
	Eresidae	*Stegodyphus mimosarum*					1022	1022						0	1022	25
	Linyphiidae	*Gnathonarium dentatum*			12			12						0	12	
	Sabaconidae	*Sabacon cavicolens*		8	13			21						0	21	
	Mecysmaucheniidae	*Mecysmauchenius segmentatus*						0			16			16	16	

Diplopoda	Spirostreptidae	*Doratogonus sp. GG-2003*	11	17				28						0	28	
	Xystodesmidae	*Cherokia georgiana*	73302	75294	24	25		148645		11				11	148656	3
Entognatha Collembola	Hypogastruridae	*Hypogastrura sp. CB02*	14					14		43			45	88	102	
	Katiannidae	*Sminthurinus bimaculatus*						0					27	27	27	
	Neanuridae	*Morulina verrucosa*						0		30				30	30	
	Sminthuridae	*Sminthuridae env sample*		20	37			57		16	251	33	52006	52306	52363	
	Sminthuridae	*Sminthurides aquaticus*						0					288	288	288	
	Entomobryidae	*Orchesellides sp. 9688-2 FZ-2013*			23			23	151	101	56			307	330	
Insecta	Cecidomyiidae	*Camptomyia corticalis*						0	1165					1165	1165	24
	Chironomidae	*Acricotopus lucens*						0	88					88	88	
	Culicidae	*Aedes aegypti*					9857	9857						0	9857	13
	Culicidae	*Uranotaenia lowii*					7	7		3		25	42	70	77	
	Culicidae	*Coquillettidia perturbans*					124		335	2				337	337	
	Culicidae	*Ochlerotatus atropalpu*					8		141					141	141	
	Formicidae	*Notostigma carazzii*	22			118			260	6517	40	3	3	6823	6823	14
	Formicidae	*Linepithema humile*	11	23	27	97			14	16				30	30	
	Mycetaeidae	*Mycetaea subterranea*						0			75			75	75	
		**Total Arthropods**	**73396**	**75362**	**438**	**2356**	**11018**	162570	**3143**	**6897**	**3663**	**795**	**54273**	68771	231341	
Mollusca Gastropoda	Cochlicopidae	*Cochlicopa lubrica*						0		18315	14			18329	18329	11
	Hermaeidae	*Aplysiopsis minor*						0		52				52	52	
	Otinidae	*Otina ovata*						0		28				28	28	
Rotifer Bdelloidea	Adinetidae	*Adineta vaga*	54	56		36		146					12	12	158	
Eutardigrada	Macrobiotidae	*Paramacrobiotus richtersi sp. NG-2008*						0				11		11	11	
Fungi																
Ascomycota																
Ascomycota_IS	Ascomycota_IS	*Slimacomyces isiola*						0	55					55	55	
Dothideomycetes	Pleosporales Fam_IS	*Anguillospora furtiva*			11			11						0	11	
	Teratosphaeriaceae	*Capnobotryella sp. MA 3612*						0	60					60	60	
Eurotiomycetes	Herpotrichiellaceae	*Herpotrichiellaceae sp. F-6*						0	121					121	121	
Lecanoromycetes	Ochrolechiaceae	*Ochrolechia szatalaensis*						0	123					123	123	
Leotiomycetes	Cudoniaceae	*Spathularia velutipes*						0	71					71	71	
	Hemiphacidiaceae	*Sarcotrochila macrospora*						0	3895					3895	3895	17
Saccharomycetes	Saccharomycetaceae	*Candida palmioleophila*	54	49	622	37		762	3040	31	47		131	3248	4010	16
	Saccharomycetaceae	*Saccharomyces cerevisiae*					315	315			13			13	328	
	Saccharomycetales_unc	*Saccharomycetales_unc*						0	82					82	82	
Sordariomycetes	Ophiocordycipitaceae	*Ophiocordyceps robertsii*			15			15	55					55	70	
	Sordariomycetes_IS	*Verticillium alfalfae VaMs.102*			519			519	217					217	736	
	Stachybotryaceae	*Myrothecium sp. 7C50*		14				14						0	14	
Ascomycete sp. He1C	Ascomycete sp. He1C	*Ascomycete sp. He1C*	11			31		42						0	42	
Ascomycete_unc	Ascomycete_IS	*Ascomycete_unc*						0	1014					1014	1014	
Basidiomycota																
Agaricomycetes	Hydnaceae	*Sistotrema resinicystidium*						0	463					463	463	
Basidomycota_unc	Basidomycota_unc	*Basidomycota_unc*						0	82					82	82	
Microbotryomycetes	Sporobolomycetaceae	*Bullera miyagiana*						0	455					455	455	
Moniliellomycetes	Moniliellaceae	*Moniliella acetoabutans*						0			23			23	23	
Tremellomycetes	Cuniculitremaceae	*Kockovaella thailandica*						0	112					112	112	
	Tremellaceae	*Cryptococcus aureus*						0	129					129	129	
	Tremellales_IS	*Tremellales_unc*						0	3579		19			3599	3599	18
Chytridiomycota															0	
Chytridiomycetes	Chytridiales_IS	*Chytridiales_unc*						0				12		12	12	
	Spizellomycetaceae	*Rhizophlyctis rosea*						0				22		22	22	
	Spizellomycetales_unc	*Spizellomycetales_unc*						0	148			633		781	781	
Chytridiomycota_unc	Chytridiomycota_unc	*Chytridiomycota_unc*				73	9610	9683	9447		17	23980		33444	43127	8
Glomeromycota															0	
Archaeosporomycetes	Ambisporaceae	*Ambispora fennica*						0				17		17	17	

Zygomycota																
Entomophthoromycetes	Ancylistaceae	*Conidiobolus brefeldianus*			17			17	88					88	105	
	Ancylistaceae	*Conidiobolus coronatus*						0	252					252	252	
Cryptomycota Rozellidea	Rozellidea_LKM11_IS	*Rozellidea_LKM11_unc*						0	18051		9285	19706		47042	47042	7
Other Fungi	Fungi	*Other fungi*	29		20			49	116			9		125	173	
		**Total Fungi**	**93**	**64**	**1204**	**141**	**9925**	11427	**41655**	**31**	**9404**	**44379**	**131**	95600	107027	
Opisthokonta Flagellates - *Choanozoa*																
Choanoflagellatea	Codonosigaceae	*Monosiga_unc*					173	173			14			14	188	
	Codonosigaceae	*Monosiga ovata*						0			136			136	136	
	Codonosigaceae	*Sphaeroeca_unc*					2528	2528						0	2528	21
	Salpingoecidae	*Salpingoecidae_unc*	17562	17180	136			34878						0	34878	9
	Choanoflagellatea_IS	*Choanoflagellatea_IS*						0				126		126	126	
Ichthyosporea	Pseudoperkinsidae	*Ichthyophonida LKM51_unc*	4331	3328	71581	3126	11198	93564	12103	330	299	5140	157	18029	111593	4
	Pseudoperkinsidae	*Pseudoperkinsidae_unc*					173	173			701	85		786	959	
	Rhinosporideacae	*Rhinosporideacae_uc*					479	479						0	479	
Heterotrophic amoeba Cercozoa																
Granofilosea	Clathrulinidae	*Hedriocystis_unc*					59	59						0	59	
Protists																
Bigyra - Bicoecea	Bicosoecaceae	*uncultured bicosoecid*					100	100						0	100	
Cryptophyta Goniomonadaceae	Goniomonadidae	*Goniomonas sp. SH-3*						0			341			341	341	
Cryptophyta Cryptophyceae	Cryptomonodaceae	*Cryptomonas reflexa*						0		289		45		334	334	
Miozoa - Colponemea	Colponemidae	*Colponema sp. Vietnam*			20			20						0	20	
Plants and Green Algae																
Magnoliids	Aristolochiaceae	*Aristolochiaceae env samp*						0	82					82	82	
Polypodiopsida	Dicksoniaceae	*Dicksoniaceae env samp*						0			18	4	8	30	30	
Chlorophyceae	Dunaliellaceae	*Polytomella*	4	4	52			60		14			20	34	94	
